# Soft Foils and Plastic Stoppings

**Published:** 1894-06

**Authors:** C. E. Francis

**Affiliations:** New York


### SOFT FOILS AND PLASTIC STOPPINGS.[1]

BY DR. C. E. FRANCIS, NEW YORK.

MANY years ago, long before dentistry had reached its present advanced status, two substances only were in general use for stopping cavities in teeth,—viz., soft gold- and tin-foils. The process of annealing the former, with a view of developing its property of cohesion, as was first demonstrated to our profession by the late Professor Arthur, was at that time unknown. Fillings of either of these materials were introduced on a like principle, and both kinds of foil were similarly prepared for the purpose. The most common method of preparation was to cut the foil into strips, from three to six to the sheet, and roll or twist into ropes or coils, or fold into ribbons. One end of the coil (usually half- or full-length of the sheet) was carried with the foil-carriers or "tweezers" to the mouth of the cavity, and pressed in. With a good-sized "plugger," attached to a strong ivory handle, the foil was worked to the distal part of the cavity, or to a point where the best anchorage could be obtained, and forced "home." The entire strip or coil, thus worked and securely packed, was followed by another coil, and so continued until every portion of the cavity was loaded, and the cavity walls made as secure as possible to prevent leakage.

Although the foils then in use possessed to a certain degree properties of cohesion, little or no dependence was placed on a perfect union of the different layers as they were packed against each other. Sharp, smooth, flattened points, of various angles, massed the folds together, and wedge-shaped indentations were made on the surface of the filling in the direction of the cavity walls, which in turn were also filled, and this continued until the filling became so thoroughly impacted as to resist further effort to puncture its surface or to add to it more filling-material. Occasionally the foils were formed into loose pellets of various sizes, especially for larger fillings, and used in connection with the folds or ropes; and in some instances the strips of foil were made into cylinders by rolling them around smooth broaches. The cylinders were carried to the tooth with one end of each resting upon the floor of the cavity and the other ends projecting from the entrance. These were also crowded together against the cavity walls and against each other, until the cavity was full and no more could be added. The pro-

---

[1] Read before the New York Odontological Society, March 20, 1894.

jecting ends of the cylinders were then forced against the cavity-margins and towards the centre until a hard smooth surface was obtained.

Although the rubber dam, now in such common use and so wonderfully efficacious in insuring absolute protection from oral secretions, was then unheard of, many of our old-time dentists succeeded in introducing fillings that did most efficient service, suggesting a degree of skill and patience on the part of their builders worthy our ardent admiration.

It was my good fortune, when a student, many years ago, to examine quite a number of soft gold-foil fillings in the mouth of a Boston gentleman, which a few years previously had been inserted by that grand old New England dentist Dr. Joshua Tucker. They were large, and some of them contour fillings, with smooth, brilliant surfaces. They were decidedly beautiful, and to my young eyes a feast so rich as to incite my earnest efforts, with a hope for similar achievements. Many other operators of that period were equally successful, despite their old-fashioned instruments and limited conveniences.

Before plastic stoppings were in vogue, tin-foil was used in many instances as a substitute for gold, especially in large cavities; probably with a view to economy; but it fulfilled its mission in a most reliable manner. A large contour filling in a devitalized superior bicuspid, introduced by the late Dr. Riggs, of Hartford, over forty-five years ago, stood securely for two decades, a monument of dental skill, and even after the surrounding walls of enamel had, from severe service, broken away, the solid lump of tin did effective duty as a masticator, boldly meeting its occluding neighbor in its work.

Dr. Lord, of this city, at a meeting of this Society several years ago, instanced a case where a tin-foil filling, in the mouth of a lady, which was inserted sixty years before, was yet in good condition. Some time ago I received a call from one of my early patients, who exhibited a very large tin-foil filling in a superior molar which I inserted twenty-five years before, and which was still in an excellent state of preservation.

Before the era of amalgam as a tooth-stopping, tin-foil was extensively employed, but since the advent of plastic stoppings it has been crowded nearly out of use.

Amalgam was introduced in this country by the famous Crawcour brothers in the year 1833, and a new departure in the way of tooth-fillings was thus established. The material was so soft and pliable, and could be manipulated with so little effort on the part of the operator, and inserted with such comparative ease to the patient, that it became a most inviting and exceedingly popular filling with many dentists then in practice, and at the present time is even more extensively employed. Always, however, there have been certain objections to this substance of sufficient weight to restrict its use with many operators, and although some of the objections are based on prejudice, or are purely imaginary, others are so well founded as to cause hesitation on the part of many cautious ones who at times greatly feel the need of some such filling-material. Efforts are continually in progress to overcome these objections and to perfect this filling, but whether or not they prove successful, it is evident that amalgam has "come to stay," and not likely to be entirely dispensed with. In many cases it has certainly proved of great value, and there are certain conditions where no other material will answer so good a purpose.

Gutta-percha stopping appeared before the public in 1848, and met with decided favor, especially among those who decried amalgam. It was first produced by the late Dr. Asa Hill, of Norwalk, Conn., and, after subsequent improvement in its manufacture, became much used, and proved an excellent stopping for buccal surfaces of posterior molars and other cavities not much exposed to attrition. The best gutta-percha stopping ever placed in market was manufactured by a Mr. Bevin, of Boston, a practical experimenter and worker in rubber goods at the factory of the Goodyear Company. Unfortunately for our profession, and I may also add for the community at large, Mr. Bevin died without ever having disclosed the secret of his formula; and although good preparations of gutta-percha have since been produced, none have ever quite equalled for toughness and durability the famous "Bevin's stopping."

Gutta-percha stopping, if well prepared and carefully introduced, will preserve the cavity walls of a tooth a number of years, and even though the surface wears away, the remainder still clings to the walls with a wonderful tenacity. These stoppings, however, expand more or less in the mouth, and are liable to burst asunder frail cavity walls; and in deep cavities, where pulps are nearly exposed, this same expansion of the filling crowds forcibly against the thin layer of dentine covering the pulp, causing irritation and perhaps congestion to this delicate organ. But gutta-percha has its place, and is valuable in many cases, especially so as a temporary stopping for children's teeth.

Next in order of filling-materials ushered into existence were the fillings of oxide of zinc, the oxychloride antedating the oxyphosphate some fifteen or more years. Each have their special value, but the former is not much used at present. The latter, however, is extensively employed, although serving only a temporary purpose. The oxide of zinc fillings waste away readily, and require frequent renewals. They cannot be counted on as reliable for long service, especially as patients are likely to neglect having them renewed. The oxyphosphate may be used to good advantage in filling the teeth of children; also as a floor or covering to a thin layer of dentine over a nearly exposed pulp.

In the course of the dentist's practice he occasionally finds tolerably large cavities with frail chalky walls, bearing evidence of rapid decalcification. These cavities, or some of them, may have been previously treated to fillings of gold, and very likely introduced as carefully as circumstances would permit; yet, so far as the preservation of the teeth was concerned, the fillings proved decided failures. Other cavities of this character may have once been filled with oxyphosphate of zinc, but in the course of time this material had succumbed to the agents of dissolution, leaving cervical borders undermined and unprotected. Perhaps gutta-percha had for a time kept some of these cavities securely, but finally yielded to the forces of attrition, and but a fragment remained. And when amalgam had been tried, jagged and discolored margins revealed the story of leaky fillings and the mischievous intrusion of decalcifying fluids, which perhaps destroyed the vitality of the organs or nearly ruined the integrity of their structure.

In viewing such results, the question naturally comes to us, What can be done to save such delicate teeth and prevent them from going to utter destruction?

Fifty or more years ago, there would have been little hesitation by dentists of that period in coming to a decision. If the teeth were considered worth filling at all, tin-foil would undoubtedly have been chosen as the most reliable material for the purpose. And if tin-foil answered so good a purpose in primitive times, and with primitive instruments and inadequate means for protection against moisture, why may it not at the present time, under far more favorable conditions for impaction, answer as well, or even better than in former times? The peculiarity that tin possesses is its ready adaptation to the walls against which it is packed, and this is an exceedingly important point to consider. It shows less rigidity in process of manipulation than gold, consequently requires less force to condense it. It can be pressed compactly against the cervical margins, and worked securely against delicate cavity-walls with little fear of crushing their borders. It does not "bulge," nor show evidences of expansion or contraction,—faults frequently attributed to amalgam. It does not wash out, as do stoppings of oxide of zinc, nor does it swell or wear away like gutta-percha.

The fact that many gold fillings in proximal cavities have failed to preserve their cervical edges has induced a number of dentists to adopt the plan of packing such borders with tin and completing the fillings with gold. It is claimed by those who have been in the habit of combining these two metals that greater safety is thus secured, or that the fillings are less likely to become undermined at these particularly vulnerable points,—the cervical borders. This seems a very rational conclusion, and possibly a combination of tin and gold, used in this manner, will insure the safety of such cavities in bicuspids and molars better than if filled entirely with gold. Yet objections have been made to such a combination. Fillings of this sort in time become much discolored, and to a certain extent the tin exhibits a tendency to oxidize,—much more so than where tin is used alone. This is probably due to a galvanic action of the two metals thus packed together. But leaving out the gold, the tin may be so firmly forced into a cavity as to present a pretty dense surface, as has been repeatedly demonstrated by many of the old-time operators. It was not for lack of density that the use of tin-foil was abandoned by those who formerly employed it, or that its use became so limited; neither for fear of oxidization, nor for lack of confidence in its preservative properties. It was given up simply because other materials came into use that were more easily and quickly manipulated; and viewing the matter also from a financial stand-point, tin-foil fillings were discarded because they were considered the least profitable of all others.

In former times, tin was viewed by all parties as a cheap filling-material, and individuals who had their teeth filled with it seemed to take it as a matter of course that the cost of such fillings would be very much less than if gold had been used. They did not take into account the time employed in preparing cavities or introducing the stopping, but rather based their ideas on the relative value of the two metals. Dentists, on the other hand, finding that it required the same length of time to prepare cavities for tin as for gold, and nearly as much time to introduce it, consequently reasoned that it would be more pleasing to their patients and more profitable to themselves to make use of the "precious metal." Then, too, "gold" has a *royal* sound, and sometimes there is much in a name.

But, gentlemen, we seem to have turned a leaf and reached another era in the history of tin stopping. A new form of this metal has recently been introduced that possesses a property which bids fair to rival all other materials in special cases. I refer to tin shavings, as recently exhibited at the Brooklyn meeting by Professor Darby, of Philadelphia, specimens of which were presented to the members of this Society at the January meeting by Dr. Perry. These shavings are produced by fixing a block of refined tin in a turning-lathe, from which may be pared, with a suitable chisel, long, thin, narrow strips. These strips or shavings form a most admirable filling-material. They can be packed and condensed with comparatively little effort, have a decided cohesive property, and the metal is so pliable that it can be worked snugly against the walls of cavities. In every respect, so far as has been tested, it seems preferable to the tin-foil of former periods. Although quite cohesive, it offers little resistance to the force of the filling instrument. It can be worked by hand-pressure or under the influence of the mallet, and when finished presents a beautiful surface.

Within a few weeks, or since the last meeting of this Society, a still greater improvement has been made in the manufacture of tin filling, in the form of cohesive tin-foil. The discovery made, or idea suggested, by Professor Darby, and so ably demonstrated by him, that tin in a state of absolute purity possessed a most decided cohesive property, induced one of our oldest and best-known manufacturers of gold-foil (Mr. Kearsing) to experiment with the metal with a view of producing a foil possessing this much-desired property of cohesion. The object sought has been attained, and we have now at our command a filling-material which, if discreetly used, has no superior for preserving cavity-walls, or protecting them from further action of decalcifying agents. This new foil, although cohesive, possesses no rigidity, as is the case with cohesive gold foil, but can readily be carried to every part of a cavity with no show of resistance. It is far more easily conveyed to the cavity than the shavings produced by Professor Darby, and can be consolidated with hand-pressure instruments or any sort of mallet; yet where the mallet is used, the blows should be decided, but not too rapid. The same form of instruments may be employed as for gold fillings, yet possibly the serrations of some of the condensing-points might be a trifle sharper.

The fillings can be built or extended to any desired contour, and when well condensed their surfaces are sufficiently hard to receive a smooth and brilliant polish, much like fillings of gold. They may be finished with keen-edged sickles, corundum wheels, and paper disks, and polished with buffs and burnishers.

In using tin it is not well or wise to lay too much stress upon the fact that it is an inexpensive metal and only fit for a cheap filling. If the mind of the operator is possessed with this idea, he may lack the required inspiration for effective results. The same degree of care should be exercised in preparing cavities, trimming their margins, sterilizing with antiseptics, and thoroughly drying with bibulous paper and warm air, as if gold were to be used. Also the same precautions observed for preventing moisture from coming in contact with the filling.

In all cases, when we have decided to employ tin-foil, we should feel that we are doing the best possible thing for the benefit of our patients, and that the preservation of the tooth or teeth under treatment is the main object to be considered.

In commending pure tin as a valuable agent for filling a certain class of cavities, I do not wish to be understood as disparaging the use of other materials in common use for filling teeth. They all have their places, and each has its peculiar merit. I simply claim that cases frequently present themselves for our care and attention where tin in its present improved form will serve a far better purpose than any other material which has as yet been brought to notice for securing safety to cavity-walls or cavity-margins, and preserving cavities in which they are impacted a greater length of time, without further deterioration or loss of tooth-structure.

In my paper at this time I have avoided making reference to the various forms of gold employed as tooth-stoppings, and dilating upon their comparative merits, deeming this a fit subject to be brought before the Society on some future occasion.

